# Clinical differences between children with asthma and rhinitis in rural and urban areas

**DOI:** 10.25100/cm.v50i1.4196

**Published:** 2019-03-30

**Authors:** Andrés Fandiño-Losada

**Affiliations:** Universidad del Valle, School of Public Health, Faculty of Health, CISALVA Institute, Cali, Colombia.

**Dear Editors:**

**Figure 1:**
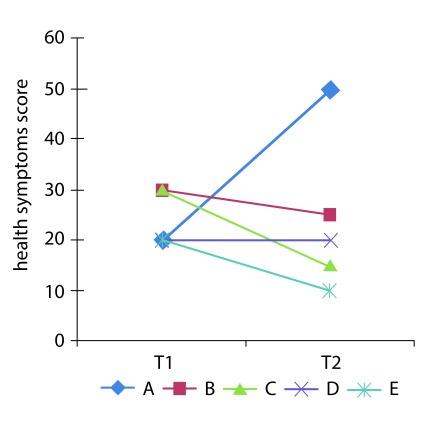
Hypothetical follow-up of a health symptoms score of five subjects.

In their article, Sánchez, *et al* ^1^ have reported about an interesting study on asthma and rhinitis symptoms among children in urban and rural Colombian areas, describing that the control of symptoms, over time, is more difficult among children in urban areas. Their article has the methodological advantage of having done a prospective follow-up of pediatric patients with asthma and rhinitis; nevertheless I have some questions:

1) The sample size calculation is not clear. The authors mentioned the prevalence of asthma and rhinitis in the urban areas and their corresponding error, but they did not use these parameters in the sample size calculation. On the other hand, the authors argued that the main outcome was comparing the treatment of asthma and rhinitis between urban and rural areas; thus readers could assume that the measure of effect was the difference of the Asthma Control Test (ACT) score between both areas; but the authors did not clearly explain it. Furthermore, it is not clear why the ratio of urban/rural subjects is 1.57; is the ratio observed in the study health centers? In this line, it seems that the appropriate sample size calculation must have been the difference of means between two independent populations, although the authors did not report any ACT effect size based on previous studies. Thus, I have estimated the means difference (as the effect size) given the power (80%) and the study sample size (urban= 201 and rural= 128) provided by the authors; using Stata® 14.2 (*power twomeans* command). In this manner, the detectable effect size would be 3.5; which is higher than the ACT effect size estimated in the Sánchez, Sánchez and Cardona’s article (i.e.: 3.0), leading to an underpowered study, at least for the cross-sectional estimates. In this manner, what was the appropriate sample size calculation?

2) The research was designed as a follow-up study with four assessments, at 3, 6, 9 and 12 months, but the statistical analyses were done using a cross-sectional approach at each measurement without taking into account the multilevel nature of the repeated (or longitudinal) measures of each patient and without the baseline adjustments of the outcome scores. The cross-sectional analyses are not appropriate for determining within-subject’s longitudinal patterns; for example, Figure 1 shows the hypothetical follow-up of a health symptoms score of five subjects, with two assessments over time (T=1 and T=2). The estimated cross-sectional mean at each time is the same (24 points), but individual persons’ trajectories (i.e. the lines) show different patterns: some subjects improve and others worsen over time. In this manner, it is important to emphasize that in the Sánchez, Sánchez and Cardona’s article, the monthly measures of each single patient are nested (or clustered) into each individual, which constitutes a longitudinal multilevel structure^2^ . Nowadays, there are several parametric and non- parametric statistical approaches for dealing appropriately with this kind of longitudinal data analysis: i.e. follow-up of patients with repeated measures of the outcome variables over time ^3^ ^,^ ^4^. The current longitudinal data analyses techniques have the advantage of allowing for the analyses of incomplete and unbalanced longitudinal data: i.e. data with missing measurements (under the MCAR or MAR missing data assumptions), attrition and/or different assessment moments^5^ ^,^ ^6^. Furthermore, nowadays these longitudinal data analysis, with mixed regression models or Bayesian approaches, have been implemented in several statistical software packages^4^. These techniques allow dealing not only with normal continuous outcomes, but also with non-normal continuous, dichotomous and polytomous categorical outcome variables ^6^. Furthermore, in Sánchez, et al. ^1^, Figure 3, the 12-months pharmacotherapy comparisons between urban and rural children did not adjust for the baseline values of the corresponding pharmacotherapy scores, which were different between urban and rural children. Thus, the estimated differences at the 12-months follow-up could be explained, instead, by those baseline scores. Finally, due to the research design is an epidemiological observational study, it is necessary to perform analyses adjusting for the confounding variables which are conceptually related with the outcome variables ^7^ ^,^ ^8^, because children’s urban or rural residences were not randomly allocated. In this manner, what are the effects of the urban environment on the pharmacotherapy scores and the symptoms of asthma and rhinitis over time, after performing the appropriate statistical analyses (i.e. multipleand repeated measures ^3^ ^,^ ^4^ regression models)?

3) When the design of a research is a longitudinal study with repeated measures, and its data are appropriately analyzed with up-to-date statistical methods, this situation allows dealing with smaller sample size given the efficiency of the longitudinal data analysis methods, which improves when adding more measurements per subject ^4^. Sample size calculation guidelines, for studies with repeated measures, have been addressed by Guo, Logan, Glueck and Muller ^9^. Did you take into account these sample size calculations in your study?

4) Neither the location nor the health care level of the study centers are clear; nor are the criteria to select them. For example, if children of rural areas with the worst symptoms levels are referred to higher complexity health centers different to those in the study, it could be a selection bias which could affect the study findings ^7^ ^,^ ^10^. Thus, which are the health care levels and locations of the study centers and which are the related potential biases?
