# Supplementary material for: Clinical differences between children with asthma and rhinitis in rural and urban areas
Source: Colomb Med (Cali). 2019 Mar 30;50(1):46–51. doi: 10.25100/cm.v50i1.4196 (PMC6536041; doi:10.25100/cm.v50i1.4196)
Supplement: Related texts

# Reply: Authors Response: Clinical differences between children with asthma and rhinitis in rural and urban areas

**Authors:** Jorge Sanchez, Andres Sánchez, Ricardo Cardona

**Affiliations:** 1 Universidad de Antioquia, Group of Clinical and Experimental Allergy, IPS Universitaria. Medellin, Colombia.; 2 Fundación para el Desarrollo de las Ciencias Médicas y Biológicas. Cartagena, Colombia; 3 Corporacion Universitaria Rafael Nuñez, Medicine Department, Cartagena, Colombia

Dear Editors:

We greatly appreciate the interest shown in the article "Clinical differences between children with asthma and rhinitis in rural and urban areas", which we hope will be one of several future articles that we intend to carry out in the study cohort. To the questions generated by the reader, one is focused on the calculation of the sample size, while the other two questions are focus in the method of analysis, and the reader suggests, it could be more robust.

Regarding the sample size, we describe that infant asthma in urban areas of Medellin was 11% and rhinitis 23%, according to previous studies. There is no data available for the rural area. We note that with a confidence level of 95%, a power of 80% and a sample size error of 0.5%, the sample size was calculated; estimating 201 children for the urban area and 128 for the rural area. Finally, we recruited and were able to continue for a year, a total of 248 children from the urban area and 134 from the rural area. The complaint of the reader, is focus that the more appropriated technique would be "*… the appropriate sample size calculation must have been the difference of means between two independent populations, although the authors did not report any ACT effect size based on previous studies*." First, we fully agree with the reader that for this type of design, the study lost power by the form of sample size calculation. We did not find studies with the urban and rural ACT tests in the studied population, which made it impossible to obtain these parameters to perform the sample size calculation by the technique "power two means" (difference of means of two independent groups). ). As we noted in the article, we do not have previous data in the rural population that allow us to infer the precise prevalence of asthma in this area; as we also noted in the article the prevalence in Colombia of asthma in the general urban population is 11%, there are also data that indicate that in the child population (less than 12 years) it is around 23%; if we work with these two prevalence where we assume that the highest in children is for the urban population and that possibly the lowest corresponds to what happens in the rural population ^1^ and assuming the parameters; alpha 0.05, power 0.80, delta 0.12 rural prevalence 0.11 vs. urban prevalence 0.23, a sample size of 306 is required; 153 for each group; in the urban area we had the availability of 201 children and in the rural area we made 128, which is close to the desired. Therefore we consider that we fulfill the expectation according to the mathematical formula used (chi-squared test comparing two independent proportions).

Regarding the following two questions of the reader that are focus in the design of the study and the analysis of the data; it is important to clarify that although a follow-up was carried out over time, for this study we wanted to focus in compared two groups (rural and urban) specially after 12 months of clinical treatment. We agree with the reader that a longitudinal analysis of repetitive measures would be appropriate and would provide additional interesting information for the effect of changes in the tests over time (e.g. ACT), however, for this study, we wanted to focus the results and the discussion, in compare these measures in two moments clinically relevant for the doctor; In the first consultation, the doctor sends a treatment and in the follow-up can make adjustments according to the clinical response; usually after one year if patient have a good clinical control, begins the process of dismantling the therapies. That’s why we preferred to highlight the final moment versus the initial moment. We recognized that the information in the intermediate times (3 months and 6 months) could be analyze in a longitudinal way, and in this form, we could evaluate the effect, but it contributes less for the objective that we planted, that was to compare if there were differences in the groups studied, especially at the end of the study, presumably due to the medical interventions carried out.

Again, we thank the reader for their pertinent suggestions that put in their real dimension the results of our research and that will serve us for the next analyzes that we will carry out with this population.

## References

[1] Riedler J, Braun-Fahrländer C, Eder W, Schreuer M, Waser M, Maisch S, Carr D (2001). Exposure to farming in early life and development of asthma and allergy: a cross-sectional survey. Lancet.

# Letter: Diferencias Clínicas entre niños con asma y rinitis en áreas urbanas y rurales

**Estimados Editores:**

En el artículo de Sánchez, *et al*.^1^, reportaron un interesante estudio sobre síntomas de asma y rinitis en niños en áreas urbanas y rurales de Colombia; identificando que el control de los síntomas es más difícil para los niños de las áreas urbanas. Ese artículo tiene la ventaja metodológica de haber realizado un seguimiento prospectivo de los pacientes pediátricos; sin embargo tengo varias preguntas sobre el mismo:

1) El cálculo del tamaño de la muestra no es claro. Los autores mencionaron las prevalencias de asma y rinitis en las áreas urbanas, con su error correspondiente, pero ellos no utilizaron esos parámetros para el cálculo del tamaño de la muestra. Por otro lado, los autores argumentaron que el desenlace (o variable resultado) principal del estudio fue comparar el tratamiento de asma y rinitis entre los niños de las áreas urbanas y rurales; entonces, los lectores pueden asumir que la medida de efecto utilizada fue la diferencia en el puntaje de la Prueba de Control del Asma (en inglés, Asthma Control Test - ACT) entre las dos áreas; pero los autores no explicaron este punto claramente. Adicionalmente, no es claro por qué el cociente entre los sujetos de las áreas urbanas/rurales es 1.57: ¿Es éste el cociente encontrado en las instituciones de salud del estudio? En consecuencia, parece que el cálculo para el tamaño de la muestra apropiado debe haber sido la diferencia de medias (o promedios) entre dos poblaciones independientes, aunque los autores no reportaron ningún tamaño de efecto de la prueba ACT basado en estudios previos. De esta forma, recalculé la diferencia de medias (como medida de efecto), utilizando el poder (80%) y el tamaño de muestra (urbano= 201 y rural= 128) reportado por los autores; usando Stata® 14.2 (comando *power twomeans*). De esta forma, el tamaño del efecto mínimamente detectable debería ser 3.5; el cual es mayor que la medida del efecto sobre el puntaje del ACT, estimada en eeste estudio ^1^ (es decir: 3.0); resultando así un estudio con bajo poder estadístico, al menos para las estimaciones transversales. Teniendo en cuenta estas consideraciones, ¿cuál fue el procedimiento de cálculo del tamaño de la muestra utilizado para planear este estudio?

2) La investigación se diseñó como un estudio de seguimiento con cuatro mediciones (o valoraciones) después de transcurridos 3, 6, 9 y 12 meses; pero los análisis estadísticos fueron realizados siguiendo un abordaje transversal en el momento de cada medición, sin tener en cuenta la naturaleza longitudinal (o multinivel) de las medidas repetidas de cada paciente y sin ajustar tampoco por los puntajes de línea de base de cada paciente. Los análisis transversales no son apropiados para determinar los patrones longitudinales intra-sujetos; por ejemplo, la Figura 1 muestra el seguimiento hipotético de un puntaje de síntomas de salud para cinco sujetos, con dos mediciones a lo largo del tiempo (T=1 y T=2). Si se calcula la media de forma transversal para cada tiempo de medición, el valor es el mismo (24 puntos), pero las trayectorias individuales de cada persona (es decir, las líneas) muestran diferentes patrones de síntomas: algunos sujetos mejoran y otros empeoran con el paso del tiempo. De esta forma, es importante enfatizar que en el artículo de Sánchez et al.^1^, las medidas mensuales de cada paciente están anidadas (o aglomeradas) en cada individuo, lo cual constituye una estructura multinivel longitudinal ^2^. Hoy en día, existen diversos abordajes estadísticos (paramétricos y no paramétricos) para analizar apropiadamente este tipo de datos longitudinales, es decir, datos a partir del seguimiento de pacientes con mediciones repetidas de las variables desenlace a lo largo del tiempo ^3^^,^^4^. Las técnicas actuales para el análisis de datos longitudinales (o repetidos) tienen la ventaja de permitir realizar los análisis estadísticos cuando existen datos longitudinales incompletos o no balanceados, es decir, datos con valores perdidos (cumpliendo los supuestos MCAR o MAR de datos perdidos, por sus siglas en inglés), o con pérdidas a lo largo del seguimiento o datos con diferentes momentos de medición ^5^^,^^6^. Complementariamente, hoy en día las funciones para realizar los análisis de datos longitudinales, usando modelos de regresión mixtos (de efectos fijos y aleatorios) o de abordajes bayesianos, han sido implementadas en diferentes paquetes estadísticos informáticos ^4^. Estas técnicas permiten analizar no solo variables desenlace continuas con una distribución normal, sino también variables resultado del tipo continuas no-normales, dicotómicas y categóricas polinomiales ^6^. Por otro lado, en la Figura 3 de Sánchez et al.^1^, para las comparaciones de la farmacoterapia a los 12 meses, entre niños del área urbana vs. área rural, no se realizó ningún ajuste (o control) de los valores de línea de base de los puntajes de farmacoterapia correspondientes, cuyos valores eran diferentes entre los niños del área urbana vs. rural. Consecuentemente, las diferencias resultantes entre estas áreas a los 12 meses de seguimiento podrían explicarse, en su lugar, por las diferencias en los puntajes de línea de base entre las dos áreas y no por la residencia de los niños en un área específica. Finalmente, debido a que el diseño de la investigación es un estudio epidemiológico observacional, entonces es necesario realizar análisis estadísticos que ajusten por (o controlen) las variables confusoras que están relacionadas, teórica o conceptualmente, con las variables desenlace del estudio ^7^^,^^8^, porque el lugar de residencia de los niños (urbano o rural) no fue asignado al azar. Teniendo en cuenta la explicación anterior, ¿cuáles son los efectos del ambiente urbano sobre los puntajes de tratamiento farmacológico y de síntomas de asma y rinitis, a lo largo del tiempo, después de realizar los análisis estadísticos apropiados (es decir, modelos de regresión múltiple ^7^ y de medidas repetidas ^3^^,^^4^)?

**Figura 1:** Seguimiento hipotético de un puntaje de síntomas de salud de cinco sujetos.

3) Cuando el diseño de una investigación es un estudio longitudinal con medidas repetidas y los datos son analizados adecuadamente utilizando los métodos estadísticos actualizados, esta situación permite utilizar tamaños de muestra más pequeños, dada la eficiencia de los métodos de análisis de datos longitudinales. La eficiencia mejora cuando se agregan más mediciones para cada sujeto de investigación ^4^. Las guías para el cálculo del tamaño de muestra para estudios de medidas repetidas han sido abordadas por Guo, Logan, Glueck y Muller ^9^. De esta forma, ¿se tuvieron en cuenta estas guías para el cálculo del tamaño de muestra en este estudio?

4) En el artículo no se especifica ni la ubicación ni el nivel de atención de las instituciones de salud del estudio; ni tampoco se explican los criterios para haber elegido esas instituciones. Esta situación tiene varias implicaciones: Por ejemplo si los niños del área rural con los peores niveles de síntomas fueron referidos a centros de atención diferentes a los centros elegidos en esta investigación, entonces pudo haber ocurrido un sesgo de selección, el cual afecta la validez de los hallazgos del estudio ^2^^,^^10^. Teniendo en cuenta lo anterior, ¿cuál era el nivel de atención y la ubicación de las instituciones de salud del estudio y que sesgos potenciales se pudieron haber presentado dada la selección de esas instituciones?

# Reply: Respuesta de los autores: Diferencias Clínicas entre niños con asma y rinitis en áreas urbanas y rurales

Estimados editores: 

Apreciamos enormemente el interés mostrado por el artículo "Diferencias clínicas entre niños con asma y rinitis en áreas rurales y urbanas", el cual esperamos sea uno de los futuros artículos que pretendemos llevar a cabo en la cohorte del estudio. A las preguntas generadas por el lector, una se enfoca en el cálculo del tamaño de la muestra, mientras que las otras dos preguntas se enfocan en el método de análisis. El lector sugiere que podría ser más sólido.

Con respecto al tamaño de la muestra, describimos que el asma infantil en las áreas urbanas de Medellín fue del 11% y la rinitis del 23%, según estudios anteriores. No hay datos disponibles para el área rural. Notamos que con un nivel de confianza del 95%, una potencia del 80% y un error de tamaño de muestra del 0.5%, se calculó el tamaño de la muestra; estimando 201 niños para el área urbana y 128 para el área rural. Finalmente, reclutamos y pudimos seguir por un año, un total de 248 niños del área urbana y 134 del área rural. La solicitud del lector, se centró en que la técnica más apropiada sería "... el cálculo del tamaño de muestra apropiado debe haber sido la diferencia de medias entre dos poblaciones independientes, aunque los autores no informaron ningún tamaño de efecto de ACT basado en estudios anteriores". Primero, estamos totalmente de acuerdo con el lector en que para este tipo de diseño, el estudio perdió potencia por la forma de cálculo del tamaño de la muestra. No se encontraron estudios con las pruebas ACT urbanas y rurales en la población estudiada, lo que hizo imposible obtener estos parámetros para realizar el cálculo del tamaño de la muestra mediante la técnica "potencia de dos medias" (diferencia de medias de dos grupos independientes). Como señalamos en el artículo, no tenemos datos previos en la población rural que nos permitan inferir la prevalencia precisa del asma en esta área; Como también señalamos en el artículo, la prevalencia de asma en Colombia en la población urbana en general es del 11%, también hay datos que indican que en la población infantil (menos de 12 años) es de alrededor del 23%; si trabajamos con estas dos prevalencias donde asumimos que la más alta se encuentra en niños de la población urbana y que posiblemente la más baja corresponda a lo que sucede en la población rural ^1^ y asumiendo los parámetros; alfa 0.05, potencia 0.80, delta 0.12 prevalencia rural 0.11 vs. prevalencia urbana 0.23, se requiere un tamaño de muestra de 306; 153 para cada grupo; en el área urbana tuvimos la disponibilidad de 201 niños y en el área rural reclutamos 128 niños, que está cerca del numero deseado. Por lo tanto, consideramos que cumplimos con las expectativas de acuerdo con la fórmula estadisitica utilizada (prueba de chi cuadrado que compara dos proporciones independientes).

Con respecto a las siguientes dos preguntas del lector que se centran en el diseño del estudio y el análisis de los datos; es importante aclarar que aunque se realizó un seguimiento a lo largo del tiempo, para este estudio quisimos centrarnos en comparar los dos grupos (rurales y urbanos), especialmente después de 12 meses del tratamiento clínico. Estamos de acuerdo con el lector en que un análisis longitudinal de medidas repetitivas sería apropiado y proporcionaría información adicional interesante para el efecto de los cambios en las pruebas a lo largo del tiempo (p. ej., ACT); sin embargo, para este estudio, quisimos enfocar los resultados y la discusión en comparar estas medidas en dos momentos clínicamente relevantes para el médico; en la primera consulta, el médico envía un tratamiento y en el seguimiento puede realizar ajustes de acuerdo con la respuesta clínica. Generalmente después de un año, si el paciente tiene un buen control clínico, comienza el proceso de desmantelamiento de las terapias. Es por eso que preferimos resaltar el momento final frente al momento inicial. Reconocimos que el análisis de la información en los tiempos intermedios (3 meses y 6 meses) fue de forma longitudinal y, que de esta forma, podíamos evaluar el efecto, pero contribuye menos al objetivo que planteamos, que fue comparar si hubo diferencias en los grupos estudiados, especialmente al final del estudio, probablemente debido a las intervenciones médicas realizadas.

Nuevamente, agradecemos al lector sus sugerencias pertinentes que ponen en su dimensión real los resultados de nuestra investigación y que nos servirán para los próximos análisis que realizaremos con esta población
